# Exploring Food Preferences as a Pre-Step for Developing Diabetes-Friendly Options in Adults with Diabetes and Prediabetes

**DOI:** 10.3390/foods13203276

**Published:** 2024-10-16

**Authors:** Sungeun Choi, Jihee Choi

**Affiliations:** Department of Family, Nutrition, and Exercise Sciences, Queens College, The City University of New York, Flushing, NY 11367, USA; jihee.choi@qc.cuny.edu

**Keywords:** taste, food preference, comfort food, diabetes, prediabetes

## Abstract

Given the low compliance with healthy eating among patients with diabetes, personalized dietary plans incorporating their food preferences are urgently needed. However, few studies have explored the food preferences of adults with diabetes or prediabetes (AdDMP). We aimed to examine taste and food preferences among AdDMP, comparing them by sex, age, and weight status. A total of 415 AdDMP completed the survey via Amazon Mechanical Turk in 2023 (53% women, 47% men; 20–70 years old). Food/taste preferences were measured using Likert-type scales for six taste-cluster food groups, as well as basic tastes/spicy flavor. Open-ended questions assessed comfort, favorite, and least favorite foods, which were then categorized into five groups. Independent *t*-tests, analysis of variance, and Tukey–HSD were performed to compare outcomes across the groups. Men, the 41–70-year-old group, and the obese group regarded warm food as a comfort food more than in other comparable groups, while women and the 20–30-year-old group tend to prefer fruits and vegetables. Additionally, men expressed a significantly higher preference for salty-/umami-/fat-tasting foods compared to women. These findings underscore the need to align dietary expectations with reality for AdDMP. Future research should focus on strategies to accommodate their preferences within a healthy eating framework.

## 1. Introduction

Diabetes mellitus is a chronic, metabolic disease that affects how the body processes food and energy, and can have serious health consequences [1]. Diabetes is a major public health problem that can cause heart disease, stroke, kidney failure, and vision loss [2]. According to the National Diabetes Statistics Report 2022 [3], 11.3% of the US population has diabetes, while 38% have prediabetes. This leads to a staggering total cost of $413 billion in medical expenses and lost productivity in the United States [3]. Unlike many other chronic conditions, diabetes management relies heavily on self-care, with patients playing a pivotal role in maintaining their health [4,5]. Healthy eating stands out as a crucial aspect of self-care for individuals with diabetes [6,7]. However, adherence to healthy eating in patients with diabetes has been shown to be low, particularly regarding the management of calorie intake [5,8]. Low adherence or non-adherence to dietary guidelines among patients with diabetes can lead to the aforementioned diabetes-related complications and, in the worst cases, result in death [2,5,8]. Given this situation, there is an urgent need for effective strategies to improve adherence to healthy eating habits and better manage calorie intake among patients with diabetes.

In light of the importance of self-care in diabetes management, a personalized approach becomes pivotal. Recent shifts in healthcare culture underscore the significance of person-centered care, emphasizing the need to truly understand each individual [9]. Building upon this ethos, Van Haitsma and colleagues introduced the Preference-Based Model of Care, which integrates various theories of human motivation and determination rooted in Maslow’s hierarchy of needs [10,11]. According to these theories, when individuals’ needs align with their preference-based values and goals, they achieve optimal growth and functioning [9,10]. Consequently, preferences serve as a vital measurement tool, operationalizing abstract constructs such as individual needs, values, and aspirations [12]. Evidence from previous studies underscores the positive impact of honoring patients’ preferences in healthcare delivery [9,10,12]. While previous studies have explored various aspects of diabetes and prediabetes management, including the progression of prediabetes to diabetes [13], the effectiveness of shared decision-making in diabetes prevention [14], and general preferences and adherence to treatment [15,16], no current research specifically addresses how a preference for certain food or taste can influence blood glucose control in patients with diabetes or prediabetes. Understanding these preferences is crucial, as dietary habits play a significant role in managing blood glucose levels and overall diabetes management [6,7]. Applying this principle to diabetes management, we recognize a better way to improve adherence to dietary regimens. Rather than imposing dogmatic dietary patterns, success lies in incorporating an individual’s food/taste preferences [12]. By doing so, we can address the needs of consumers with diabetes or prediabetes, who constitute almost half of the adult population in the U.S. [3], and promote their adoption of sustainable dietary practices.

Food decisions are influenced by a complex interplay of economic, social, and biologically determined behavioral variables, including food availability [17,18]. Among these factors, taste and other sensory properties of foods emerge as critical determinants in the selection process [18,19,20,21]. Previous studies recommend assessing taste perception and food preference, as the optimal combination of “beyond liking” and “liking” measures can better predict food consumption in practical applications for dietary intervention [18,19,20,21,22]. For instance, elevated sweet preferences correlate with greater consumption of added sugars and sweet foods, while heightened fat liking is associated with increased fat intake [18,19,23]. People with diabetes often prioritize short-term food enjoyment over long-term health goals, such as weight loss or glycemic control [24,25]. Furthermore, their taste impairments [26,27,28,29,30] and poor eating habits [5,24] further complicate adherence to a diabetes-specific diet. Consequently, they express dissatisfaction with current dietary recommendations for diabetes management and encounter substantial barriers when striving for healthier meals [5,26,27,28,29,30].

Extracting personal preferences is undoubtedly crucial, yet it remains a formidable task [31,32,33,34]. Existing questionnaires, often comprising extensive lists of individual food items, attempt to assess food preferences while considering cultural nuances [35,36,37,38,39]. However, these instruments fall short in comprehensively capturing multifaceted food preferences [39]. Moreover, the preference for a specific food item can be influenced by its preparation and cooking methods [35,39]. Therefore, a holistic approach—one that encompasses cultural experiences and considers all sensory attributes—is essential for a comprehensive understanding of individual food preferences. Recognizing the significance of patient preferences within dietary regimens, our study delved into taste and food preferences. Specifically, we employed open-ended questions to explore comfort foods, favorite dishes, and least preferred foods. This study represents the first attempt to comprehensively assess taste- and food-preference profiles in this specific population. By examining these preferences, we aimed to uncover potential differences based on sex, age, and body weight status. Our findings can provide valuable insights for the food and foodservice industries, enabling them to develop more tailored and effective strategies. These strategies could include creating personalized meal options and targeted marketing campaigns for different demographic groups, such as gender, age, and weight groups with specific dietary needs. Efforts to develop diabetes-friendly food options can ultimately contribute to improved public health by enhancing compliance with dietary recommendations for individuals with diabetes or prediabetes, conditions that have become highly prevalent in recent times.

## 2. Materials and Methods

### 2.1. Participants

A total of 442 participants, aged 18 to 70 and diagnosed with diabetes or prediabetes, were recruited through Amazon Mechanical Turk (M-Turk) (Amazon Mechanical Turk, Inc., Seattle, WA, USA) across the United States. To determine eligibility for the study, we implemented an additional screening process. Participants were asked if they had been diagnosed with prediabetes or diabetes by a healthcare professional. Furthermore, individuals who scored above 5 on the ‘Prediabetes Risk Test’ by the American Diabetes Association (ADA) and the Centers for Disease Control and Prevention (CDC) [40] were considered eligible for inclusion. Those who did not meet these criteria were excluded from the study. A power analysis was conducted to determine the sample size required to detect a medium effect size (*f* = 0.25) in a one-way analysis of variance, using G*Power (version 3.1.9.7, 2020, Department of Psychology, Universität Düsseldorf, Düsseldorf, Germany) [41,42]. Given the variability in food preferences and the anticipated effect sizes from previous literature [43,44], a sample size of 280 was calculated to achieve a power of 0.95 with a type I error probability of 0.05. To account for potential dropouts or incomplete responses, we aimed to recruit a larger number of participants, resulting in a final sample size of 442.

### 2.2. Survey

This study used cross-sectional data from a web-based survey. The survey conducted via M-Turk website between November and December 2023. M-Turk was selected because it provides a demographically and geographically diverse population, which can provide a sample more representative than traditional survey methods [45,46,47]. Additionally, previous studies proved that M-Turk generates reliable and psychometrically valid results for behavioral and experimental research [48]. Upon successful completion of the survey, $5 was deposited into each participant’s M-Turk account as an incentive for participation. The Qualtrics survey tool was used to develop a web-based survey. The study protocol was reviewed and approved by the City University of New York, University Integrated-Institutional Review Board (IRB#2023–0756), and informed consent was obtained from each subject prior to their participation in the study.

The following questions were included in the survey: demographic questions; weight and height questions; food preference items, “How much do you like following tasting food groups?” for 6 taste–cluster food groups (neutral, sweet/sour, sweet/fat, fat, salty/umami/fat, bitter) that was used to describe dietary intakes on the base of taste qualities [49] using a 7-point hedonic scale with the following answers: Extremely dislike = 1, Dislike = 2, Slightly dislike = 3, Neutral = 4, Slightly like = 5, Like = 6, Extremely like = 7; taste satisfaction items, “Generally, how much of each of the following tastes/flavors do you want to have in your food to satisfy your taste buds?” using a 5-point Likert-type scale with the following answers: Not at all = 1, A little = 2, Somewhat = 3, Much = 4, Very much = 5; open-ended questions for comfort, favorite, and least favorite foods. In the comfort food question, the provided definition of comfort food states that it may be consumed either to relieve negative psychological effects or to enhance positive feelings [50]. These open-ended responses were categorized into five groups (meat, seafood, fruits and vegetables, cold complex food, and warm complex food) for statistical analyses. In this study, participants were asked about their sex assigned at birth, and only binary sex categorization (woman/man) was used.

### 2.3. Statistical Analysis

Chi-square tests were utilized to compare categorical values and independent samples *t*-tests and one-way analysis of variance (ANOVA) were used to compare continuous variables of participants. Tukey–HSD tests were performed for multiple comparisons of continuous variables. The statistical assumptions for each test were checked and met. For all analyses, a *p* value < 0.05 indicated statistical significance. Statistical analyses were performed using SPSS software for Windows (version 29.0, 2022, IBM Corp, Armonk, NY, USA).

## 3. Results

A total of 442 participated in the survey. After data screening to remove surveys with missing and invalid answers, the final dataset was composed of 415 adults with diabetes or prediabetes. Descriptive characteristics for the participants are displayed in Table 1. Of the respondents, 53% were women and 81.4% were White. In this study, participants ranged in age from 20 to 70 years. Of these, 52.3% (*n* = 217) were between 20 and 30 years old, 30.8% (*n* = 128) were between 31 and 40 years old, and the remaining 16.9% were 41 years or older (Table 2).

**Figure 1:**
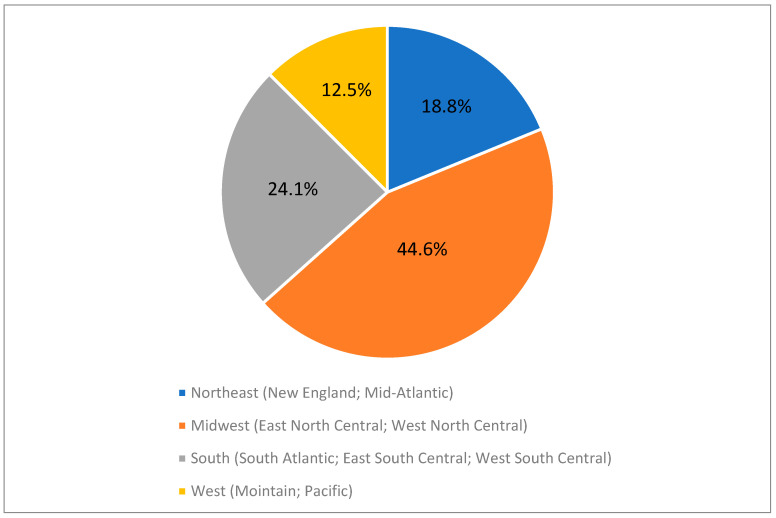
Regional distribution of survey participants’ (*n* = 415) residences in the United States.

Most participants were college graduates (58%), and Christianity was the dominant religious affiliation (83.9%, including Protestantism and Roman Catholicism). The annual household income was evenly distributed across the categories, except for the highest (>$120,000, 3.6%) and lowest (<$20,000, 1.9%) income categories. Marital status was predominantly married (86.3%), and the majority lived in the Midwest region of the United States (44.6%) (Figure 1). The most prevalent dietary restriction among participants was a vegetarian diet (encompassing vegans, pesco-vegetarians, and other vegetarians), with 33.8% adhering to it. This was closely followed by Halal, at 31.8% (Table 1). We conducted statistical analyses to determine the influence of race, educational level, religion, and income on the results. However, no significant effects were found for any of these factors, likely due to the skewed distribution of the data.

In response to an open-ended question about comfort food, participants reported a diverse range of items, from fried chicken to ice cream. The top comfort foods, each accounting for more than 3% of responses, included chicken (12.6%, including fried chicken at 2.2%), burgers (9.2%), seafood (7.5%), casseroles (6.5%), cakes or pastries (including doughnuts, 6.3%), vegetables (including French fries, 6%), rice dishes (such as paella or biryani, 5.5%), beef/lamb/mutton/goat (4.6%), pizza (4.3%), soup (4.3%), pasta (3.9%), sandwiches (3.9%), cheese (3.4%), and ice cream (3.1%). In Table 2, we present the results of responses to comfort, favorite, and least favorite food questions categorized by sex, age, and weight status. When categorizing these comfort foods, respondents consistently favored warm complex dishes like soup or casserole as their top choice. This preference remained consistent across sexes, age groups, and weight status categories (Table 2).

However, the second preferred comfort foods varied by sex, age, and weight status. Women, the youngest age group (20–30 years old), as well as those with normal and overweight status, leaned toward cold complex foods. In contrast, men, older age groups, and the obese category favored meat as their second top comfort food. Notably, there were significant differences in comfort food preferences between sexes (χ^2^ = 21.7, *p* < 0.001), age groups (χ^2^ = 28.9, *p* = 0.001), and weight status groups (χ^2^ = 38.3, *p* < 0.001) (Table 2).

For favorite food that was asked using an open-ended question, a variety of food was reported from fried chicken to sushi. The following items are the top favorite foods (>1%): chicken (36.4% including fried chicken 13%); burger (15.7%); pizza (12.8%); French fries (8.2%); seafood (6% including sushi 2.4%); soup (4.6%); noodle (2.7% including Ramen 0.5%); pork/sausage/hot dog (1.4%); fruits (1.4%). The top two items—chicken and burger—are the same in both the comfort food and favorite food groups. However, the percentages of favorite food for those items are higher than for comfort food. Like comfort foods, after grouping all favorite food items into five categories, respondents consistently reported warm complex foods such as soup or casserole as their top favorite food in total. These responses applied to both men and women, across all age groups except the 20–30-year-old group, and all weight status categories except the overweight group (Table 2). The 20–30-year-old group and overweight group reported meat as their top favorite food. Although there is no significant difference (χ^2^ = 8.6, *p* = 0.128) between sexes, men reported meat, seafood, cold complex food, and warm complex food more as a favorite food than women did, whereas women reported fruits and vegetables more than men (Table 2). There is a significant difference in favorite food distribution across age groups (χ^2^ = 35.0, *p* < 0.001). Among the two older age groups (31–40 and 41–70 years old), warm complex food was predominantly favored followed by meat as their second most preferred food. In contrast, the youngest age group (20–30 years old) primarily chose meat as their favorite, with warm complex food ranking second. Among the weight status groups (underweight, normal, and obese), warm complex food was predominantly favored followed by meat as their second most preferred food. In contrast, the overweight group primarily chose meat as their favorite, with warm complex food ranking second (Table 2).

For least favorite food that was asked using an open-ended question, a variety of food was reported from fried chicken to sushi. The following items are the top least favorite foods (>2%): vegetable such as broccoli or Brussel sprout (25% including French fries 0.7%); seafood (11.6%); fruits (9.2%); chicken (8.2% including fried chicken 4.3%); noodle (7.5% including ramen 0.5%); none (7%); pizza (4.6%); soup (4.3%); liver/organ (3.4%); burger (3.4%), pickle (2.7%); pasta (2.4%). Respondents reported fruits and vegetables as a top least favorite food in total, men, all age groups, and all weight status group except for obese group (Table 2). The most reported least favorite food in women is warm complex food and second least favorite food is fruits and vegetables (Table 2). There are significant differences in least favorite food distribution between men and women (χ^2^ = 16.8, *p* = 0.005) and the age groups (χ^2^ = 18.7, *p* = 0.044). Men reported meat, fruits and vegetables, and none more as a least favorite food than women did, whereas women reported cold complex and warm complex food more than men (Table 2). The obese group primarily selected warm complex foods as their least favorite, while fruits and vegetables ranked second. Interestingly, the underweight group ranked meat and fruits and vegetables equally as their least favorite (Table 2).

Table 3 presents the results of preference responses related to taste–cluster food groups, basic tastes, and spicy flavors, categorized by sex, age, and weight status. Notably, significant differences emerged between men and women for salty/umami/fat (*p* = 0.038) and among age groups for neutral-tasting food preferences (*p* = 0.003) (Table 3). Within the weight status groups, a significant difference was observed in the liking of neutral-(*p* = 0.002) and fat-tasting (*p* = 0.035) foods. The significance of fat-tasting food was demonstrated by ANOVA, but not by the Tukey–HSD test (Table 3). Specifically, men exhibited a significantly higher preference for salty-/umami-/fat-tasting foods compared to women, while the oldest age group (41–70 years old) favored neutral-tasting foods (Table 3).

Additionally, the obese group reported significantly lower preferences for both neutral- and fat-tasting foods compared to other weight groups. Regarding preferences for basic tastes and spicy flavors, no significant differences were found between sexes and across weight status groups. However, notable distinctions emerged between the two older age groups (31–40 and 41–70 years old, as shown in Table 3). Participants aged 31–40 expressed a desire for significantly higher degrees of bitter and fat tastes (*p* < 0.05) to satisfy their taste buds compared to those aged 41 and over (Table 3).

## 4. Discussion

Previous studies have explored various aspects of diabetes and prediabetes management, such as the effectiveness of strategies in preventing diabetes and the progression from prediabetes to diabetes [13,14], as well as general preferences and adherence to pharmacotherapy [15,16]. However, there is a lack of research specifically examining how food or taste preferences impact blood glucose control in individuals with diabetes or prediabetes. Consequently, we could not compare our findings with previous studies on food preferences in these populations and instead compared them with the general public.

Our comparison revealed that the taste and food preferences of adults with diabetes or prediabetes in our study closely resemble those of the general public, including healthy adults. We identified significant differences in the choice of comfort food (*p* < 0.001) and least favorite food (*p* = 0.005) between men and women with diabetes or prediabetes. Men reported meat, seafood, and warm complex foods more frequently as comfort foods, while women emphasized fruits, vegetables, and cold complex dishes (Table 2). Additionally, men selected fruits and vegetables as their least favorite food significantly more than women (Table 2). These results are consistent with the findings of Wansink et al. [50] for the general North American population, where women tended to report foods requiring little preparation and snacks, such as potato chips, ice cream, chocolate, and cookies, as comfort foods, while men preferred foods served by their mothers, such as soup, pizza, pasta, steak, and mashed potatoes.

Regarding taste–cluster food groups, men preferred ‘salt/umami/fat’-tasting foods significantly more than women (Table 3). The literature supports these findings, showing that men generally prefer salty and/or fatty foods more than women, who tend to prefer sweet foods [43]. The study by van Langeveld et al. also reported that men consumed a significantly larger percentage of energy from foods tasting ‘salt/umami/fat’ and ‘bitter’ compared with women (mostly *p* < 0.001) [49]. Among the age groups, there are significant differences in comfort food (*p* = 0.001), favorite food (*p* < 0.001), and least favorite food (*p* = 0.044). The oldest group (41–70 years old) tends to prefer warm complex food as a comfort and favorite foods significantly more, whereas the youngest group (20–30 years old) considers cold complex food as a comfort food significantly more than other age groups (Table 2). The results correspond well with the finding that younger people had a higher acceptance for snack-related foods than meal-related foods in the study by Wansink et al. [50]. A study by Lirette et al. [51], also found that older adults were more satisfied with their meals when served warm food. Some studies suggest the reasons that older adults may prefer warm food over cold food. Firstly, as older adults tend to have slightly lower body temperatures than younger adults [52], older adults might prefer warm food to help maintain their body temperature. Warm food can provide a sense of physical warmth and help regulate body temperature, which can be comforting. Another reason for older adults preferring warm food is their decreased sensory perception with age [53]. Since hot food produces more aroma due to carrying more airborne molecules than cold food, warm food can be more appealing to older people who have a weaker ability to smell. Furthermore, the diabetic condition itself may aggravate the sensory perception impairment [26,27,28]. Lastly, as food choices can be influenced by comfort and tradition and warm foods are often associated with comfort [54], older adults might prefer warm food for their familiarity and emotional warmth.

Among the weight status groups, significant differences were observed for neutral- and fat-tasting foods. Specifically, the obese group exhibited significantly lower liking values for neutral- and fat-tasting foods compared to other weight groups (Table 3). However, a study by van Langeveld et al. [49] found that obese individuals obtained a higher percentage of their energy from foods with ‘salt/umami/fat’ flavors compared to normal-weight individuals. The inconsistency between the self-reported preference for fat taste in our present study and the actual caloric intake from fat-rich foods in obese individuals appears to be related to discrepancies between self-reported and actual caloric intake and exercise, as reported by Lichtman et al. [55]. In their study, the authors concluded that weight-loss failure in some obese subjects results from an energy intake substantially higher than reported and an overestimation of physical activity.

The findings from this study highlight the pressing need to bridge the gap between dietary recommendations and the actual experiences of patients with diabetes or prediabetes. Doctors should recommend Medical Nutrition Therapy (MNT), which provides a systematic and evidence-based approach to managing diabetes through diet [6,7]. While its effectiveness has been demonstrated, challenges remain [56,57]. Although most diabetes guidelines suggest initiating pharmacotherapy only after implementing nutritional and physical activity lifestyle changes, this practice is not consistently followed worldwide [58,59]. One significant barrier is that many physicians lack training in nutrition interventions, making it difficult to counsel patients effectively [56]. Additionally, discussing nutrition with patients can be time consuming. Therefore, doctors should recommend counseling with trained nutritionists or diabetes educator [60].

Importantly, dietary recommendations should incorporate the food preferences of individuals with diabetes or prediabetes into personalized healthy dietary plans. Rather than imposing rigid dietary patterns, success lies in integrating an individual’s food and taste preferences into their diet. This approach does not mean endorsing unhealthy eating habits; instead, it involves finding ways to include patients’ food preferences within a healthy diabetes diet. Additional insights can be drawn from the dietary habits of regions with low diabetes prevalence. Sub-Saharan Africa, including countries like Benin and The Gambia, has some of the lowest diabetes rates globally [61]. This low prevalence can be attributed to several factors, including dietary habits [61]. Traditional diets in these regions are often high in fiber and low in processed foods and sugars. They typically include a variety of vegetables, legumes, and whole grains, which are known to help maintain stable blood sugar levels and improve insulin sensitivity [7]. These dietary habits have significant implications for the prevention and management of diabetes. By promoting a diet rich in whole, unprocessed foods and high in fiber, it may be possible to reduce the risk of developing diabetes and manage blood sugar levels more effectively in those who already have the condition [62,63,64].

This study has several limitations. Firstly, its cross-sectional design prevents the establishment of cause-and-effect relationships or the analysis of behavior over time. Secondly, relying on self-reported data introduces validity concerns. Participants may provide socially acceptable answers rather than complete honesty, and the accuracy of self-assessment may vary, affecting the capture of absolute preferences. Another drawback of self-reports is the potential limitation of closed or fixed questions, which may restrict responses. However, these limitations can be mitigated by incorporating open-ended questions. Additionally, while there are several advantages to using M-Turk, concerns about data quality have been raised due to satisficing behaviors or rushed answers [65]. To address these concerns, extra efforts were made in this study to improve data quality. In addition to directly asking ‘yes/no’ questions about study eligibility, we further maximized data quality by embedding implicit screening questions, such as the ‘Prediabetes Risk Test’ by the ADA and CDC [40]. This additional step helped verify that participants truly belonged to the target population [66]. Lastly, since the survey was conducted during winter, we could not account for temporal variations, such as differences between winter and summer. Despite the limitations of this study, it boasts several strengths. Most importantly, it represents the first examination of taste and food preferences among adults with diabetes or prediabetes, addressing a gap in research specific to this population. Another strength lies in the use of a geographically representative nationwide sample composed entirely of adults with diabetes or prediabetes. Furthermore, the study employed a multifaceted approach to assess taste and food preferences, incorporating comfort foods, taste–cluster food groups, and various taste/flavor profiles through a combination of quantitative and qualitative data.

## 5. Conclusions

The current study provides cross-sectional evidence that adults with diabetes or prediabetes exhibit distinct taste- and food-preference profiles based on sex, age, and weight status. This study also demonstrates that despite their diabetes diagnosis, individuals still have similar taste and food preferences as general population. The unchanged preferences seem to make it more difficult for them to adhere to their dietary regimen. Future research should aim to incorporate the food preferences of individuals with diabetes or prediabetes into personalized healthy dietary plans. By embracing the taste perceptions and food preferences of this group, which constitutes nearly half of the adult population in the US, the food and food service industries can develop diabetes-friendly products and menus that are highly acceptable. These expanded options can ultimately contribute to improved public health by reducing diabetes complications and the incidence of diabetes or prediabetes. Additionally, future research should include more in-depth studies, such as longitudinal studies, to examine how food preferences change over time in response to glycemic control. This approach will provide valuable insights into the dynamic relationship between diet and diabetes management, helping to refine dietary recommendations and interventions.

## Figures and Tables

**Table 1:** Sociodemographic characteristics, reported weight status and dietary restriction of 415 adults with diabetes or prediabetes.

	Total (*n* = 415)	Men (*n* = 195)	Women (*n* = 220)	*p*^1^ Value
Age, mean ± SD ^2^ (year)	34.2 ± 8.4	34.5 ± 8.6	34.0 ± 8.3	0.517
Race, *n* (% ^3^)				0.940
Non-Hispanic Whites	338 (81.4)	160 (82.1)	178 (80.9)	
Non-Hispanic Blacks	14 (3.4)	5 (2.6)	9 (4.1)	
Hispanics	37 (8.9)	18 (9.2)	19 (8.6)	
Asians	24 (5.8)	11 (5.6)	13 (5.9)	
Others	2 (0.5)	1 (0.5)	1 (0.5)	
Weight Status, *n* (% ^3^)				0.188
Underweight	42 (10.1)	23 (11.8)	19 (8.6)	
Normal	156 (37.6)	81 (41.5)	75 (34.1)	
Overweight	182 (43.9)	76 (39.0)	106 (48.2)	
Obese	35 (8.4)	15 (7.7)	20 (9.1)	
Dietary Restriction ^4^, *n* (% ^3^)				0.300
Vegan	81 (19.5)	37 (19.0)	44 (20.0)	
Pesco vegetarian	43 (10.4)	17 (8.7)	26 (11.8)	
Other vegetarian	12 (2.9)	5 (2.6)	7 (13.2)	
Kosher	87 (21)	50 (25.6)	37 (16.8)	
Halal	132 (31.8)	66 (33.8)	66 (30)	
Gluten-free	22 (5.3)	10 (5.1)	12 (5.5)	
No dietary restriction	93 (22.4)	32 (16.4)	61 (27.7)	
Education Level, *n* (% ^3^)				0.300
Less than Highschool	2 (0.5)	1 (0.5)	1 (0.5)	
Highschool or GED	38 (9.2)	23 (11.8)	15 (9.2)	
Some college	11 (2.7)	4 (2.1)	7 (2.7)	
College graduate	239 (57.5)	115 (59.0)	124 (57.5)	
Postgraduation degree	125 (30.1)	52 (26.6)	73 (30.1)	
Religion *n* (% ^3^)				0.047
No religion	15 (3.6)	7 (3.5)	8 (3.6)	
Christianity	270 (65.1)	133 (68.2)	137 (62.3)	
Roman Catholic	78 (18.8)	28 (14.4)	50 (22.7)	
Islamism	24 (5.8)	16 (8.2)	8 (3.6)	
Judaism	20 (4.8)	6 (3.1)	14 (6.4)	
Hinduism	8 (1.9)	5 (2.6)	3 (1.4)	
Annual Household Income, *n* (% ^3^)				0.003
<$20,000	8 (1.9)	3 (1.5)	5 (2.3)	
$20,000–40,000	82 (19.8)	41 (21.0)	41 (18.6)	
$40,001–60,000	119 (28.7)	64 (32.8)	55 (25.0)	
$60,001–80,000	89 (21.4)	28 (14.4)	61 (27.7)	
$80,001–100,000	102 (24.6)	56 (28.8)	46 (20.9)	
≥$120,000	15 (3.6)	3 (1.5)	12 (5.5)	
Marital Status, *n* (% ^3^)				0.714
Single	39 (9.4)	20 (10.3)	19 (8.6)	
Married	358 (86.2)	169 (86.7)	189 (85.9)	
Divorce/Separated	2 (0.5)	1 (0.5)	1 (0.5)	
Widowed	5 (1.2)	2 (1.0)	3 (1.4)	
Live in Partner	11 (2.7)	3 (1.5)	8 (3.6)	

^1^ *p* values were determined using independent *t*-test for continuous variables and *χ²* test for categorical variables, as appropriate. The level of significance was set at *p* < 0.05. ^2^ SD: Standard Deviation. ^3^ Column percentage. ^4^ A check-all-that-apply question where the column percentage may exceed 100%.

**Table 2:** Comfort foods, favorite foods, least favorite foods of 415 adults with diabetes or prediabetes.

	Total (*n* = 415)	Sex		Age			Weight Status			
		Men (*n* = 195)	Women (*n* = 220)	20–30 (*n* = 217)	31–40 (*n* = 128)	41–70 (*n* = 70)	Underweight (*n* = 42)	Normal (*n* = 156)	Overweight (*n* = 182)	Obese (*n* = 135)
Comfort Foods ^1^, *n* (% ^2^)										
Meat	75 (18.1)	42 (21.5)	33 (15.0)	32 (14.7)	29 (22.7)	14 (20.0)	3 (7.1)	27 (17.3)	38 (20.9)	7 (20.0)
Seafood	31 (7.5)	18 (9.2)	13 (5.9)	10 (4.6)	18 (14.1)	3 (4.3)	3 (7.1)	6 (3.8)	22 (12.1)	0 (0)
Fruits & Vegetables	41 (9.9)	10 (5.1)	31 (14.1)	18 (8.3)	12 (9.4)	11 (15.7)	12 (28.6)	12 (7.7)	13 (7.1)	4 (11.4)
Cold Complex	94 (22.7)	32 (16.4)	62 (28.2)	65 (30.0)	19 (14.8)	10 (14.3)	11 (26.2)	38 (24.4)	40 (22.0)	5 (14.3)
Warm Complex	167 (40.2)	89 (45.6)	78 (35.5)	88 (40.6)	49 (38.3)	30 (42.9)	13 (31.0)	71 (45.5)	65 (35.7)	18 (51.4)
None	7 (1.7)	4 (2.1)	3 (1.4)	4 (1.8)	1 (0.8)	2 (2.9)	0 (0)	2 (1.3)	4 (2.2)	1 (2.9)
		*p* < 0.001		*p* = 0.001			*p* < 0.001			
Favorite Foods ^1^, *n* (% ^2^)										
Meat	162 (39.0)	80 (41)	82 (37.3)	103 (47.5)	42 (32.8)	17 (24.3)	13 (31.0)	60 (38.5)	77 (42.3)	12 (34.3)
Seafood	15 (3.6)	9 (4.6)	6 (2.7)	5 (2.3)	6 (4.7)	4 (5.7)	1 (2.4)	4 (2.6)	9 (4.9)	1 (2.9)
Fruits & Vegetables	44 (10.6)	13 (6.7)	31 (14.1)	30 (13.8)	7 (5.5)	7 (10.0)	2(4.8)	20 (12.8)	20 (11.0)	2 (5.7)
Cold Complex	17 (4.1)	10 (5.1)	7 (3.2)	2 (0.9)	9 (7.0)	6 (8.6)	3 (7.1)	7 (4.5)	5 (2.7)	2 (5.7)
Warm Complex	176 (42.4)	83 (42.6)	93 (42.3)	76 (35.0)	64 (50.0)	36 (51.4)	23 (54.8)	65 (41.7)	70 (38.5)	18 (51.4)
None	1 (0.2)	0 (0)	1 (0.5)	1 (0.5)	0 (0)	0 (0)	0 (0)	0 (0)	1 (0.5)	0 (0)
		*p* = 0.128		*p* < 0.001			*p* = 0.671			
Least Favorite Foods ^1^, *n* (% ^1^)										
Meat	64 (15.4)	40 (20.5)	24 (10.9)	26 (12.0)	28 (21.9)	10 (14.3)	14 (33.3)	17 (10.9)	29 (15.9)	4 (11.4)
Seafood	48 (11.6)	21 (10.8)	27 (12.3)	31 (14.3)	15 (11.7)	2 (2.9)	3 (7.1)	17 (10.9)	23 (12.6)	5 (14.3)
Fruits & Vegetables	146 (35.2)	75 (38.5)	71 (32.3)	72 (33.2)	41 (32.0)	33 (47.1)	14 (33.3)	59 (37.8)	62 (34.1)	11 (31.4)
Cold Complex	18 (4.3)	8 (4.1)	10 (4.5)	9 (4.1)	4 (3.1)	5 (7.1)	3 (7.1)	7 (4.5)	6 (3.3)	2 (5.7)
Warm Complex	110 (26.5)	36 (18.5)	74 (33.6)	60 (27.6)	33 (25.8)	17 (24.3)	7 (16.7)	39 (25.0)	52 (28.6)	12 (34.3)
None	29 (7.0)	15 (7.7)	14 (6.4)	19 (8.8)	7 (5.5)	3 (4.3)	1 (2.4)	17 (10.9)	10 (5.5)	1 (2.9)
		*p* = 0.005		*p* = 0.044			*p* = 0.080			

^1^ A check-all-that-apply question where the column percentage may exceed 100%. ^2^ Column percentage. *p* values were determined using chi-square tests. The level of significance was set at *p* < 0.05.

**Table 3:** Preference values for taste–cluster food groups and satisfaction values for basic tastes and spicy flavor by 415 adults with diabetes or prediabetes.

Taste-Cluster ^3^, Mean ± SD	Total (*n* = 415)	Sex		*p* ^1^ Value	Age			*p* ^2^ Value	Weight Status				*p* ^2^ Value
		Men (*n* = 195)	Women (*n* = 220)		20–30 (*n* = 217)	31–40 (*n* = 128)	41–70 (*n* = 70)		Underweight (*n* = 42)	Normal (*n* = 156)	Overweight (*n* = 182)	Obese (*n* = 135)	
Neutral	5.3 ± 1.4	5.4 ± 1.3	5.2 ± 1.6	0.151	5.1 a ± 1.5	5.5 b ± 1.4	5.6 b ± 1.2	0.003	5.7 b ± 1.3	5.4 b ± 1.2	5.3 b ± 1.5	4.5 a ± 1.8	0.002
Sweet/sour	5.2 ± 1.4	5.3 ± 1.4	5.2 ± 1.4	0.862	5.2 ± 1.5	5.3 ± 1.4	5.2 ± 1.4	0.657	5.1 ± 1.4	5.4 ± 1.2	5.2 ± 1.5	4.9 ± 1.7	0.262
Sweet/fat	5.2 ± 1.3	5.3 ± 1.3	5.2 ± 1.4	0.306	5.1 ± 1.4	5.4 ± 1.4	5.3 ± 1.2	0.108	5.2 ± 1.2	5.4 ± 1.2	5.1 ± 1.5	5.3 ± 1.7	0.500
Fat	5.3 ± 1.4	5.4 ± 1.3	5.2 ± 1.5	0.175	5.2 ± 1.4	5.5 ± 1.5	5.1 ± 1.2	0.169	5.0 ± 1.5	5.5 ± 1.2	5.2 ± 1.5	4.9 ± 1.7	0.035
Salt/umami/ fat	5.2 ± 1.4	5.4 ± 1.3	5.1 ± 1.4	0.038	5.1 ± 1.4	5.4 ± 1.4	5.3 ± 1.1	0.171	5.1 ± 1.4	5.3 ± 1.2	5.2 ± 1.4	4.8 ± 1.7	0.253
Bitter	5.2 ± 1.4	5.3 ± 1.3	5.1 ± 1.5	0.302	5.1 ± 1.4	5.4 ± 1.4	5.3 ± 1.3	0.059	5.3 ± 1.2	5.3 ± 1.2	5.2 ± 1.5	4.9 ± 1.4	0.591
**Basic tastes & spicy flavor ^4^,** **Mean ± SD**													
Sweet	3.7 ± 1.0	3.7 ± 1.0	3.6 ± 1.0	0.421	3.6 ± 0.9	3.8 ± 1.0	3.6 ± 1.0	0.396	3.6 ± 1.1	3.7 ± 0.9	3.7 ± 1.0	3.8 ± 0.9	0.813
Salty	3.4 ± 1.0	3.4 ± 1.0	3.5 ± 1.0	0.601	3.3 ± 0.9	3.6 ± 1.1	3.4 ± 0.9	0.054	3.5 ± 1.1	3.3 ± 0.9	3.5 ± 1.0	3.7 ± 1.0	0.193
Sour	3.5 ± 1.0	3.5 ± 1.0	3.5 ± 1.0	0.886	3.4 ± 1.0	3.6 ± 1.0	3.5 ± 0.9	0.224	3.5 ± 0.8	3.4 ± 0.9	3.5 ± 1.1	3.7 ± 1.1	0.302
Bitter	3.6 ± 1.1	3.5 ± 1.0	3.6 ± 1.1	0.463	3.6 ab ± 1.0	3.7 b ± 1.1	3.4 a ± 1.1	0.036	3.5 ± 1.0	3.5 ± 1.1	3.6 ± 0.9	3.7 ± 1.1	0.432
Umami/ Savory	3.6 ± 1.0	3.7 ± 1.0	3.5 ± 1.0	0.055	3.6 ± 1.0	3.7 ± 1.0	3.8 ± 0.8	0.360	3.6 ± 0.9	3.6 ± 0.9	3.7 ± 1.0	3.6 ± 1.1	0.684
Fat	3.5 ± 1.0	3.4 ± 1.1	3.5 ± 1.0	0.855	3.4 ab ± 1.0	3.6 b ± 1.1	3.3 a ± 0.9	0.038	3.3 ± 1.1	3.3 ± 1.1	3.6 ± 1.0	3.5 ± 1.0	0.125
Spicy	3.8 ± 1.0	3.8 ± 1.1	3.8 ± 1.0	0.819	3.7 ± 1.0	3.9 ± 1.0	3.7 ± 1.1	0.099	3.8 ± 1.0	3.8 ± 1.0	3.7 ± 1.1	3.8 ± 1.0	0.949

SD: Standard Deviation. ^1^ *p* values were determined using independent samples *t* test. ^2^ *p* values were determined using one-way analyses of variance. Tukey’s Honest Significant Difference post hoc test was used for significance testing (*p* < 0.05); a, b—Mean values with different letters in a row were significantly different. ^3^ Participants were asked the following: “How much do you like following tasting food groups?” for 6 taste–cluster food groups (neutral, sweet/sour, sweet/fat, fat, salty/umami/fat, bitter) using a 7-point hedonic scale with the following answers: Extremely dislike = 1, Dislike = 2, Slightly dislike = 3, Neutral = 4, Slightly like = 5, Like = 6, Extremely like = 7. ^4^ Participants were asked the following: “Generally, how much of each of the following tastes/flavors do you want to have in your food to satisfy your taste buds?” for basic tastes (sweet, salty, sour, bitter, umami/savory) and spicy flavor using a 5-point Likert-type scale with the following answers: Not at all = 1, A little = 2, Somewhat = 3, Much = 4, Very much = 5.

## Data Availability

The raw data supporting the conclusions of this article will be made available by the authors on request.
